# The Book World of Medicine and Science

**Published:** 1899-01-14

**Authors:** 


### The Book World of Medicine and Science.

Mad Humanity. By L. Forbes Winslow, M.B, D.C.L. Pp. 450, 8vo. (London: C. A. Pearson, Limited. 1898. Price 7s. 6d.)

The general public are, no doubt, deplorably ignorant of the nature of lunacy and lamentably prejudiced in their judgments on its relations to society. We fully recognise, then, the great educational value of such works, addressed to the public, as Dr. Maudsley's. But we very seriously question the propriety of the publication of books such as the one before us. "Mad Humanity" is confessedly written for the delectation of the public—it certainly contains little of profit to the profession—and we deprecate most sincerely the publication for lay readers of details of cases whose relatives must be alive and can scarcely fail to suffer pain at the recital. It is true that no names are mentioned as a rule in this book, but in some instances the disguise is perfectly transparent. Details are given of the life of the unfortunate perpetrator of a murder that shocked London some years ago. If the details came under the author's notice in the pursuit of his profession, the publication of them here is very like a breach of confidence. In any case, considering the circumstances and the social position of the unfortunate youth, a very glaring error of taste has been committed. Much could be pardoned an author who set out to instruct the public on the position taken by the profession, and not himself alone, with regard to responsibility for crime, or who gave suitable advice to the guardians of neuropathic children, or, insisting on the advantage of early treatment of insanity, described our splendid asylums. But Dr. Winslow does none of these things; he recites piquant cases, and hints mysteriously at the men of high position, leading comedians, and so forth who have sought his advice. The scientific value of the book may be gauged when we say that in it no mention is made of one at least of the most potent causes of insanity. A judicious utterance on this point would go further to "benefit humanity"—the author's professed purpose—than any of the reproductions of insane ravings and scripts with which the book is peppered. The chapter on "Madness of Genius" demands serious criticism. To say that "it is a difficult thing to define the line that separates the babbling, drivelling idiot from the man of transcendent genius" is sensational, but unfair. The remark "that in the majority of studious men there often exists a predisposition to brain disease which may have actually existed" is as slipshod in grammar as in thought. Nor is Dr. Winslow happy in his list of mad geniuses. It is a mere abuse of terms to call Johnson and Burns "poets who became insane." Lloyd, Clare, Riddell, and Collins had nothing of the insanity of genius or the genius of insanity; they were poetasters who became insane just as might Tom, Dick, and Harry. The Verlaines and Nietzsches—true mad geniuses—find no place in Dr. Winslow's category. And we may estimate the author's literary taste by his observation that Swift's "Voyage to Liliput" is "still read by children." It is monstrous to include Scott in a list of mad geniuses. Everyone who has read Lockhart's "Life" must remember Scott's own account of how, when in the nursery, he one night hopped about the room, "caught cold," and "became afterwards paralyzed." Scott's deformity clearly, then, was the result of acute poliomyelitis and had nothing to do with any "nervous attack during inter-uterine life" as Dr. Winslow says it had. We are quite at a loss to understand what Dr. Winslow means when he says Scott's dementia was the result of "progressive flaccid degeneration of tubular neurine." Scott's dementia was post-apoplectic; the post mortem showed the apoplexy to have been due to hæmorrhage into the corpus striatum; and the "paralysis of which his brother and father died" proves nothing unless it be a family tendency to arterial degeneration, and therefore cerebral hæmorrhage. The author falls again into error when he speaks of "Abraham-men" as wandering lunatics let loose at the time of the Reformation. "Abram-men" were the sturdy vagabonds who, feigning insanity, passed themselves off as Tom-a-Bedlams, the pensionaires and discharged patients from Bethlem. We have said little of the medical side of this work. In truth it contains little that is not commonplace beyond such extraordinary assertions as that locomotor ataxy bears not the faintest resemblance to general paralysis, and that progressive muscular atrophy is entirely a spinal disease. However, it has always been the custom of "mad doctors" to fill up their pathological lacunæ with tags from Hamlet, Lear, and Othello; in this book tags abound. The frontispiece is a portrait of the author. Scattered through the book are photographs of various lunatics.

Baby Feeding; or, How to Rear Healthy Children. By a Doctor. Specially written for the wives of the working classes. (Bristol: John Wright and Co. 1898. Price 4d., or in packets for free distribution at reduced rates.)

This is an anonymous publication, but it is a capital little book and contains much common sense. Moreover, the author does not hesitate to describe in pretty forcible language the evils which he tries to combat. Addressing mothers he says, "Do you know that much of the crime committed daily on the face of this earth is largely woman's fault?" which some cynics will perhaps say is rather like a platitude. The reason, however, for this denunciation of woman is that they are responsible for "the delicate, wretched, unhealthy men and women" who inhabit this earth. "Their mothers neglected them, fed them improperly, starved them body and soul; and the result is that, at the end of this nineteenth century, man, instead of being the strongest of animals, is the weakest and feeblest being in creation." The little book which is to put right this sad condition of affairs is full of useful information upon the subject with which it deals—information as to the proper food for infants and children; the management of infancy; the weaning of the child; aphorisms as to what to do and what not to do; and an appendix full of useful recipes. If the mothers to whom it is addressed would but read it and believe its teachings much benefit to the rising generation would result.

